# Supplementary figures and images for: The Long Non-Coding RNA MALAT1 Modulates NR4A1 Expression through a Downstream Regulatory Element in Specific Cancer Cell Types
Source: Int J Mol Sci. 2024 May 18;25(10):5515. doi: 10.3390/ijms25105515 (PMC11121914; doi:10.3390/ijms25105515)

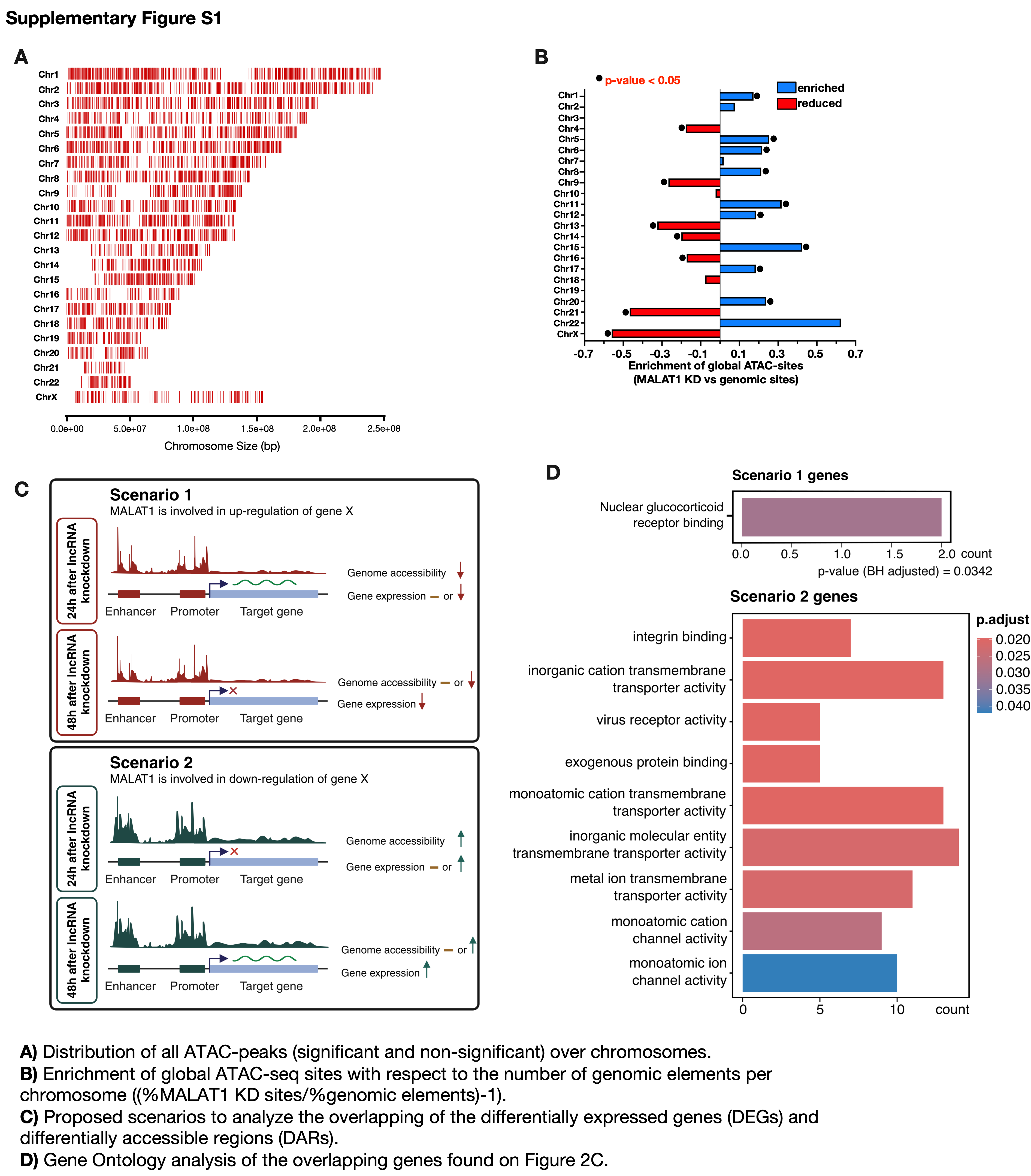

Supplement: Supplementary file 1 [file ijms-25-05515-s001.zip › Supp Fig S1.tiff]

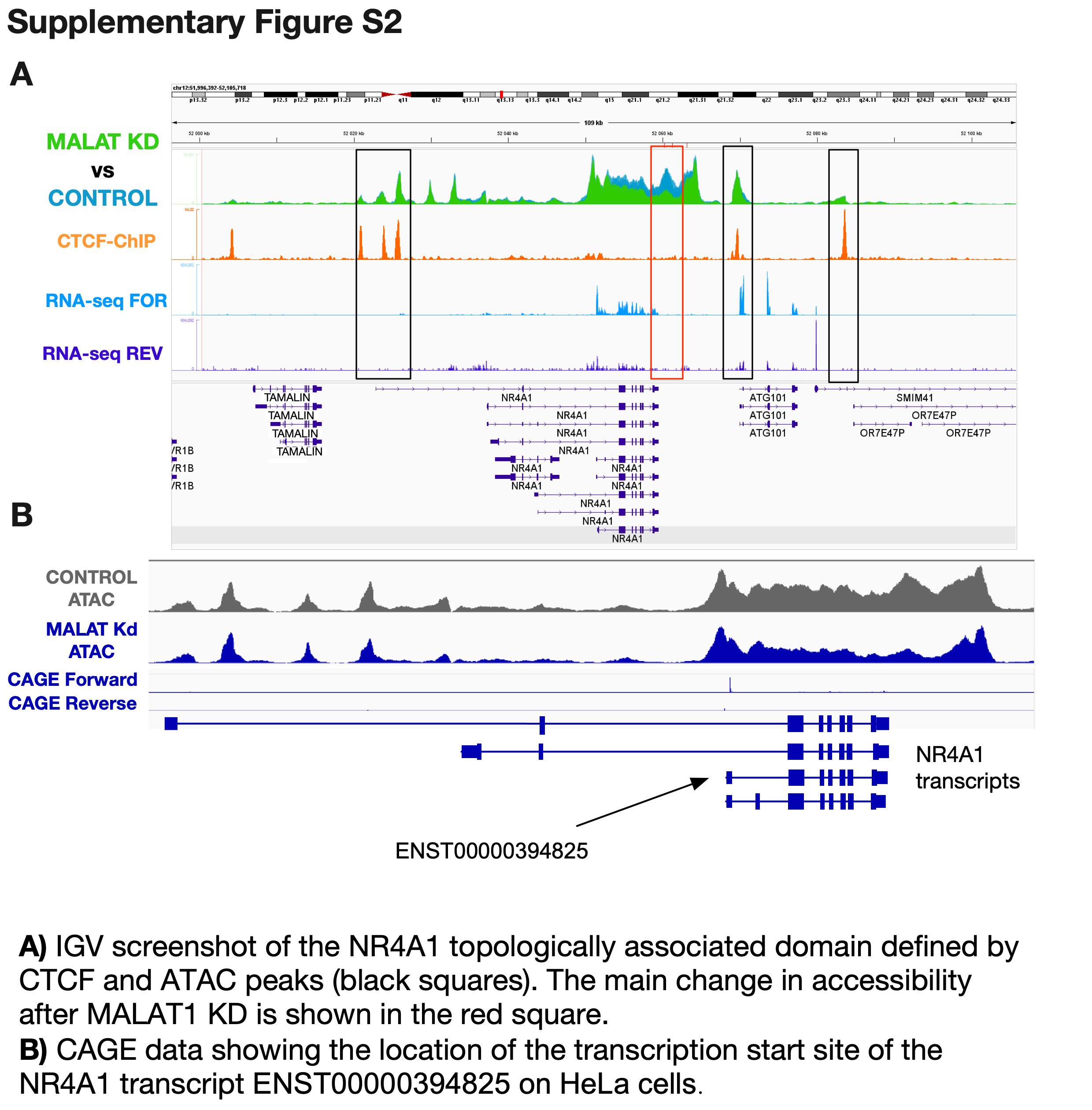

Supplement: Supplementary file 1 [file ijms-25-05515-s001.zip › Supp Fig S2.tiff]

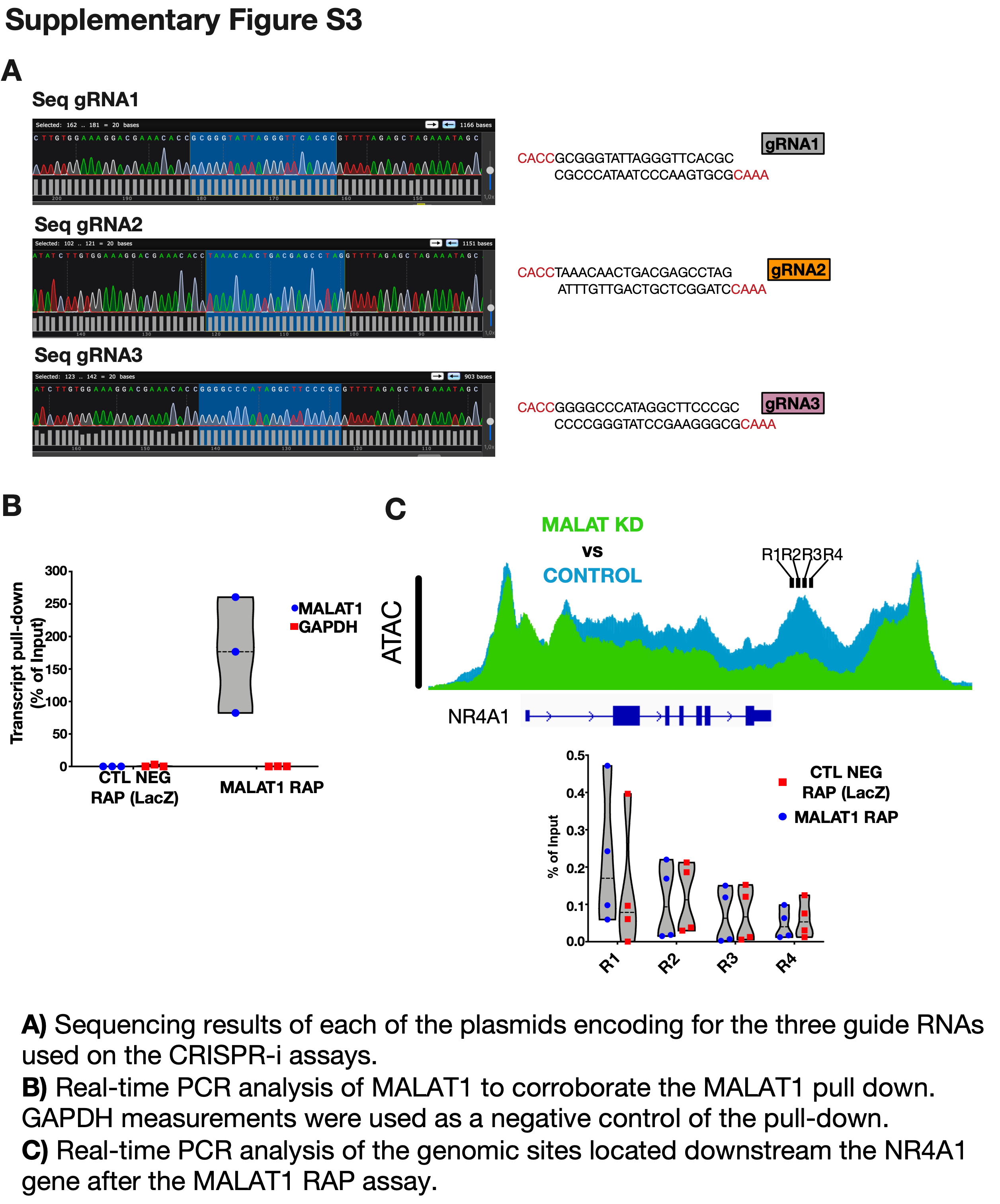

Supplement: Supplementary file 1 [file ijms-25-05515-s001.zip › Supp Fig S3.tiff]

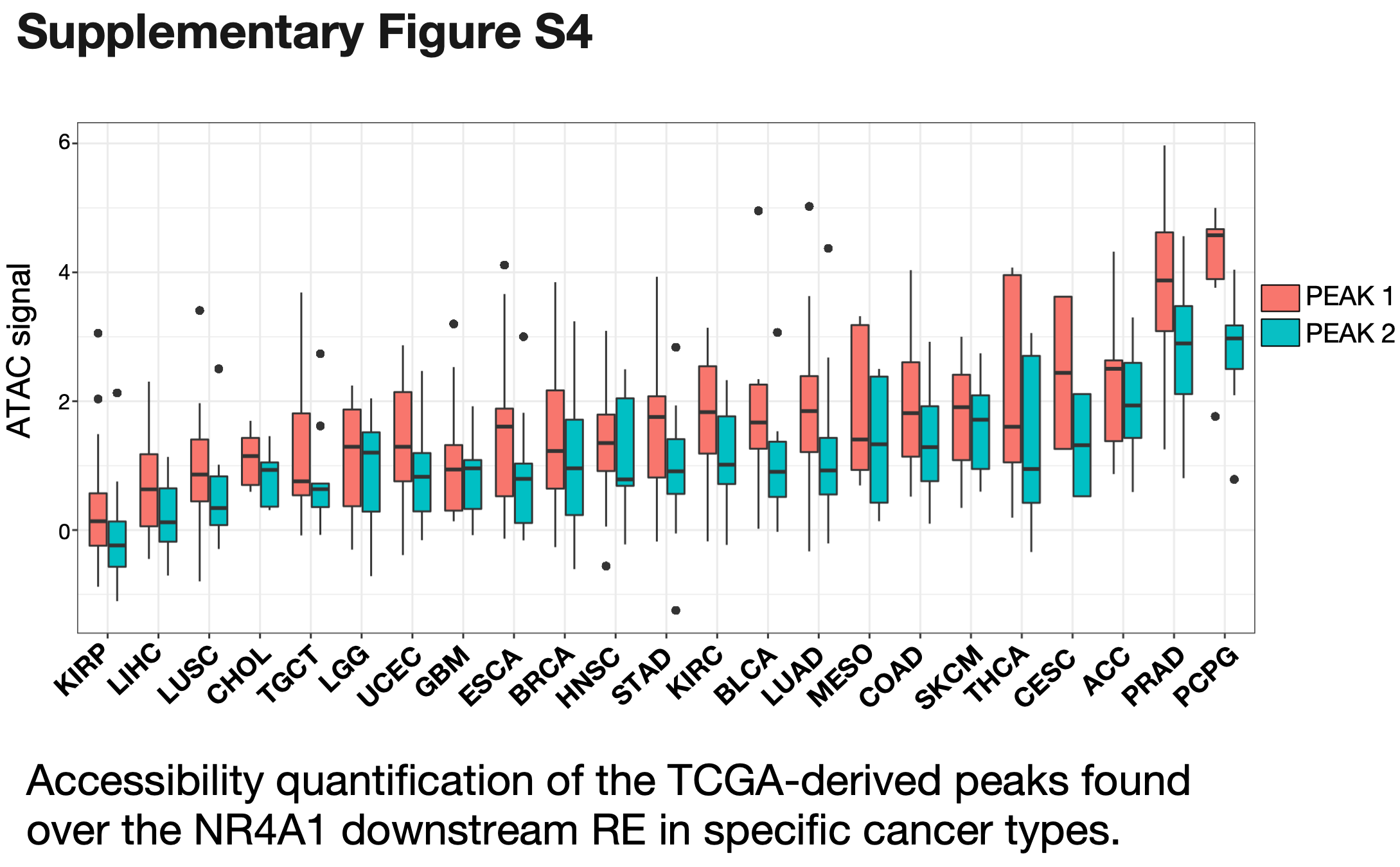

Supplement: Supplementary file 1 [file ijms-25-05515-s001.zip › Supp Fig S4.tiff]

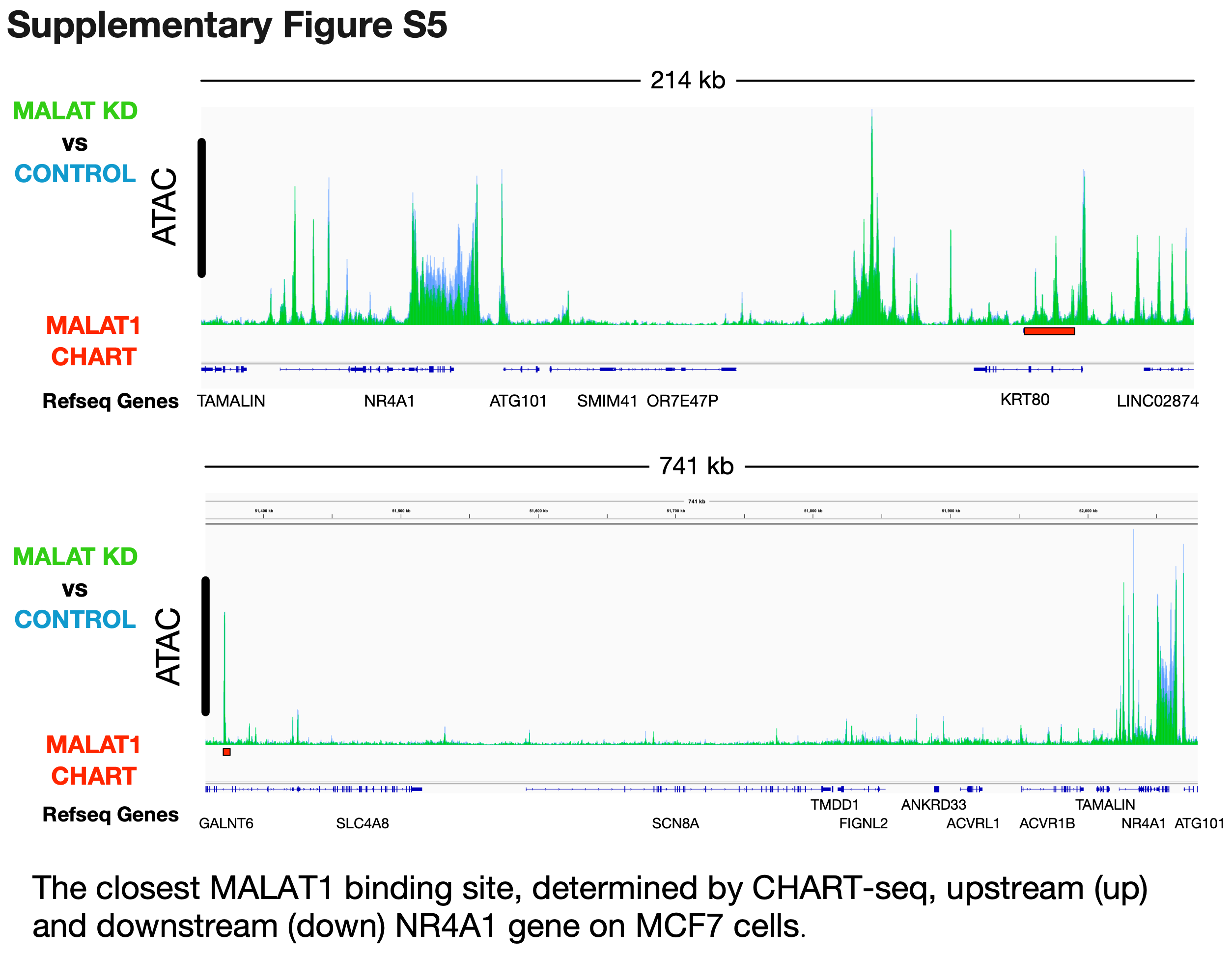

Supplement: Supplementary file 1 [file ijms-25-05515-s001.zip › Supp Fig S5.tiff]

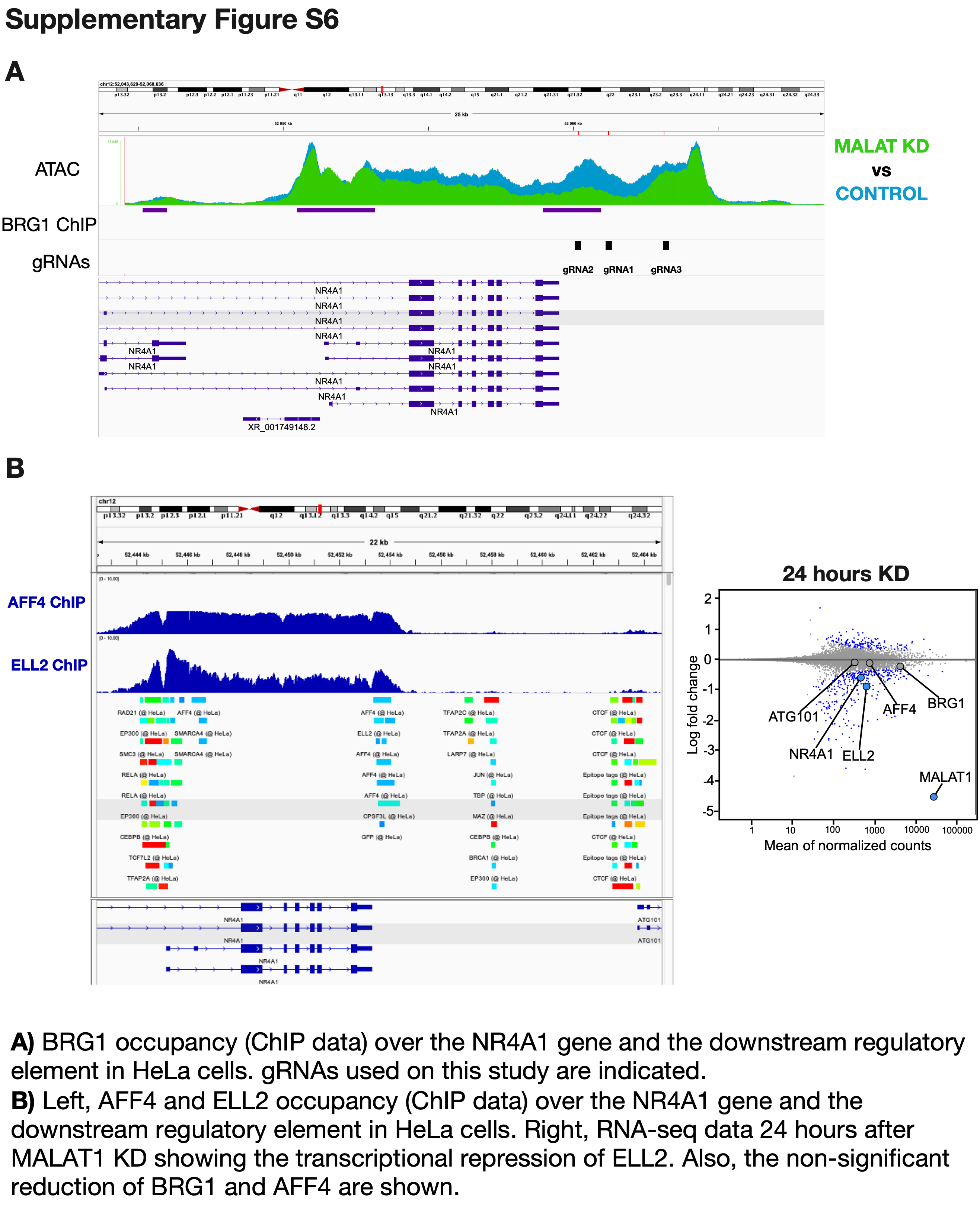

Supplement: Supplementary file 1 [file ijms-25-05515-s001.zip › Supp Fig S6.tiff]
